# Impacts of mindfulness-based interventions in people undergoing hemodialysis: a systematic review

**DOI:** 10.1590/2175-8239-JBN-2021-0116

**Published:** 2021-10-11

**Authors:** Bruno Nunes Razzera, Angélica Nickel Adamoli, Maitê Freitas Ranheiri, Margareth da Silva Oliveira, Ana Maria Pandolfo Feoli

**Affiliations:** 1 Pontifícia Universidade Católica do Rio Grande do Sul, Escola de Ciências da Saúde e da Vida, Programa de Pós-Graduação em Psicologia, Porto Alegre, RS, Brasil.; 2 Hospital de Clínicas de Porto Alegre, Serviço de Educação Física e Terapia Ocupacional de Nefrologia, Porto Alegre, RS, Brasil.

**Keywords:** Chronic Kidney Failure, Renal Dialysis, Chronic Kidney Failure, Mindfulness, Insuficiência Renal Crônica, Diálise Renal, Falência Renal Crônica, Atenção Plena

## Abstract

**Introduction:**

Chronic kidney disease (CKD) is a serious public health problem worldwide, leading to a series of physical and psychological comorbidities, in addition to costly treatments, lifestyle and dietary restrictions. There is evidence that mindfulness-based interventions (MBIs) offer complementary treatment for people with chronic illnesses, including CKD, with the aim of improving overall health, reducing side effects and treatment costs. This review aims to investigate the MBIs impact on people with CKD undergoing hemodialysis, and to identify the methodological quality of the current literature in order to support future studies.

**Methods:**

We ran searches in five databases (MEDLINE via PubMed, PsycINFO, Embase, Web of Science and Scopus) in July 2020. The papers were selected and evaluated by two reviewers independently, using predefined criteria, including the Cochrane Group's risk of bias tool and its recommendations (CRD42020192936).

**Results:**

Of the 175 studies found, 6 randomized controlled trials met the inclusion criteria, and ranged from 2014 to 2019. There were significant improvements in symptoms of anxiety, depression, self-efficacy, sleep quality, and quality of life (n=3) in the groups submitted to the intervention, in addition to physical measures such as blood pressure, heart rate and respiratory rate (n=1).

**Conclusions:**

MBIs can offer a promising and safe complementary therapy for people with CKD undergoing hemodialysis, acting on quality of life and physical aspects of the disease.

## Resumo

**Introdução:**

A doença renal crônica (DRC) é reconhecida como um sério problema de saúde pública a nível mundial, levando a uma série de comorbidades físicas e psicológicas, além de tratamentos custosos e restrições no estilo de vida e alimentares. Há evidências de que as intervenções baseadas em mindfulness (IBMs) oferecem opções complementares ao tratamento de pessoas com doenças crônicas, incluindo DRC, com o objetivo de melhorar a saúde geral, reduzir os efeitos colaterais e custos do tratamento. Esta revisão objetiva investigar o impacto de IBMs em pessoas com DRC em hemodiálise, e identificar a qualidade metodológica da literatura atual a fim de auxiliar pesquisas futuras.

**Métodos:**

As pesquisas foram realizadas em cinco bases de dados (MEDLINE via PubMed, PsycINFO, Embase, Web of Science e Scopus), em julho de 2020. Os artigos foram selecionados e avaliados por dois revisores de forma independente, utilizando critérios predefinidos, incluindo a ferramenta de risco de viés do grupo Cochrane e suas recomendações (CRD42020192936).

**Resultados:**

Dos 175 estudos encontrados, 6 ensaios clínicos randomizados estavam de acordo com os critérios de inclusão, e variaram entre os anos de 2014 a 2019. Foram encontradas melhoras significativas para os sintomas de ansiedade, depressão, autoeficácia, qualidade de sono, e qualidade de vida (n=3) nos grupos que realizaram a intervenção, além de medidas físicas como pressão arterial, frequência cardíaca e taxa respiratória (n=1).

**Conclusões:**

As IBMs podem oferecer uma terapêutica complementar promissora e segura para pessoas com DRC em hemodiálise, atuando na qualidade de vida e em aspectos físicos da doença.

## INTRODUCTION

Chronic kidney disease (CKD) is a serious public health problem worldwide^1^. People with CKD have a range of physical and psychological comorbidities^2^, in addition to facing costly treatments, lifestyle and dietary restrictions^3^^-^ 5. Dialysis is an invasive, complex and time-consuming process, leading to depression and anxiety^6^^,^ 7, sleep disorders^8^, non-compliance to dialysis^9^, and chronic pain^10^, which are associated with low quality of life and high mortality rates^11^^-^ 14.

In addition to conventional therapies for this population, complementary interventions offer new options with the aim of improving general health, reducing side effects and treatment costs^15^. The most well-established and prevalent integrative therapy for mind and body is the practice of cognitive-behavioral therapy (CBT), the one most used to treat various mental disorders, reduce stress and psychological symptoms in people with chronic diseases, due to its structure and flexible content^16^^,^ 17. Mindfulness-based interventions (MBIs) are part of the "third wave of CBT" and have been arousing interest concerning their effectiveness in clinical disorders and physical diseases, as they deal with mental and physical aspects^18^^,^ 19.

Mindfulness is characterized by paying attention to the present moment, with openness, curiosity and acceptance^20^. Mindfulness practices involve paying attention to the experience as it is in the present moment, bringing higher awareness regarding the external and internal experiences, greater cognitive and behavioral flexibility, and tolerance of unpleasantries^21^^,^ 22. Currently, there are a variety of MBI protocols for different clinical outcomes and populations^15^^,^ 23 ^-^ 26. The first protocol called mindfulness-based stress reduction (MBSR) was developed by Jon Kabat-Zinn (1990)^20^ with the aim of helping people with chronic pain and stress associated with long-term conditions; and served as the basis for the construction of other MBIs^27^.

Over the years, studies involving MBIs have shown effectiveness for a wide range of conditions, including chronic diseases^24^^,^ 28. Some systematic reviews and meta-analyses involving this theme addressed the positive effects of MBIs for chronic conditions such as: fibromyalgia^29^, somatization disorder^30^, chronic pain^31^^,^ 32, cancer^33^^,^ 34 and multiple sclerosis^35^. Other studies involving randomized controlled trials (RCTs) have shown the benefits of MBIs in people with CKD in improving quality of life^36^, depression and anxiety^37^, reducing stress^38^ and hypertension^39^.

In a recent narrative review of the effects of meditative interventions and CKD, Bennett et al. (2018)^40^ showed promisingly positive results for disorders such as anxiety, stress, depression, sleep disorders and quality of life. In addition, the authors encourage further studies on this topic, to investigate and reinforce the importance of implementing higher quality methodologies such as RCTs, use of active controls and appropriately sized samples^40^. Despite the promising effects of meditative practices and CKD, the investigation of studies using well-established MBI protocols is necessary. Thus, in order to expand knowledge on the subject, this systematic review aims to investigate the impact of mindfulness-based interventions in people with chronic kidney disease on hemodialysis, and to identify the methodological quality of the current literature in order to aid future studies.

## METHOD

This systematic review was carried out using a protocol constructed in accordance with the Cochrane Manual Recommendations,^41^ developed in accordance with the preferred report items for systematic reviews and meta-analyses (PRISMA)^42^, registered in the International Prospective Register of Systematic Reviews (PROSPERO) (CRD42020192936).

### DATA SOURCES AND RESEARCH

The search strategy was performed in the online Medical Literature Analysis and Retrieval System (MEDLINE) databases via PubMed, PsycINFO, Excerpta Medical Database (Embase), Web of Science and SciVerse Scopus (Scopus), with terms that matched the question of interest. For Embase, the search was carried out with the filter "all fields", Web of Science was filtered by "topic" and Scopus through "title, abstract and keywords". For the other databases, no filter was used. The reference lists of the included studies was also analyzed in order to identify a possible flaw in the original search. The articles included in the search had no definition of an initial period and were extracted until July 2020.

The research included keywords indexed in Health Sciences Descriptors (DECS) and Medical Subject Headings (MeSH Terms) such as: Renal Dialysis; Dialysis; Chronic Kidney Failure; Peritoneal dialysis; Chronic Kidney Failure; Nephropathies; Mindfulness; Mindfulness Meditation; Mindfulness-Based Intervention; MBI; Mindfulness Based Stress Reduction; MBSR; Mindfulness Based Cognitive Therapy; MBCT; Mindful Eating. The synonyms present in each keyword listed were included in the search. The choice of adding synonyms to the search strategy was applied in order to unify the key used in the different databases and expand the search. All potentially eligible studies were reviewed, regardless of primary outcome or language.

### STUDY SELECTION

We included only MBI studies in people with chronic kidney disease, over 18 years old on hemodialysis treatment, and written in English. Systematic reviews and meta-analyses, MBI together with other interventions, incomplete texts and themes other than the objective of the study were excluded.

### DATA EXTRACTION AND QUALITY ASSESSMENT

All citations retrieved from electronic databases were imported into an Excel spreadsheet. Two reviewers (BNR and MFR) analyzed independently and blindly, where the researchers first selected the titles and abstracts, and then the full texts, applying the inclusion and exclusion criteria established in the protocol.

Data from the included studies were independently extracted by the same two reviewers using a standardized form. The extracted data included: main author, year of publication, design, participants (including number of participants per group, mean and standard deviation of age), inclusion criteria, information about the intervention (format, frequency and duration of the program) and control group (format, frequency and duration, in the case of active controls), post-intervention follow-up evaluations, evaluation measures and main results.

The methodological quality of the included studies was independently assessed by the same two reviewers using The Risk of Bias 2 (RoB 2) tool, in its updated version for ECRs (and its variation for ECRs in crossover format)^43^. The risk of bias was categorized as "low", "some concerns" and "high" for each of the following domains: randomization process, intended intervention, missing outcome data, outcome measures, and reported outcome. Disagreements between review authors about the risk of bias in the studies were resolved by discussion, with the involvement of a third reviewer, when necessary.

## RESULTS

### STUDY SELECTION

**Figure 1:**
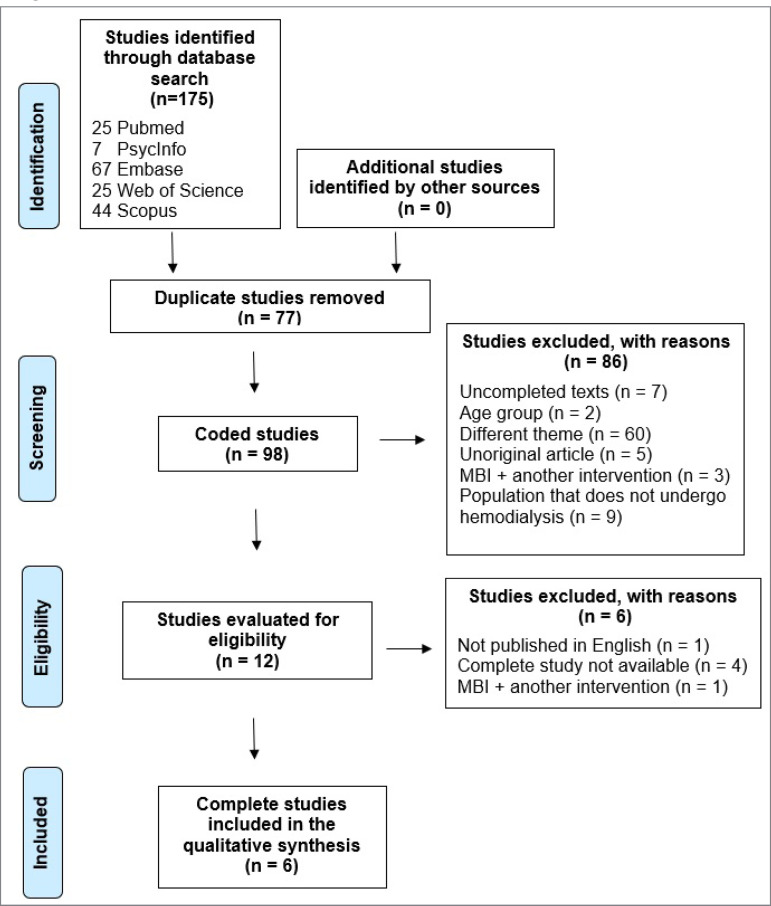
PRISMA Flowchart Note. MBI: Mindfulness-based intervention.

We found 175 potential studies in the database searches. After removing 77 duplicate studies, we coded 98 titles and/or abstracts. Following the application of the exclusion criteria (see PRISMA flowchart in Figure 1), 86 studies were excluded, and 12 studies were included. Of these, 6 studies were excluded mainly because they were abstracts published in conferences, full text in a language other than English, and other interventions in conjunction with mindfulness. As a result, 5 RCTs^36^^-^ 38 ^,^ 44 ^,^ 45and 1 crossover RCT^39^ were included for data extraction and quality assessments. The included studies were published between the years 2014 to 2019.

### STUDY CHARACTERISTICS

Table 1 describes the characteristics of the included studies, such as design, sample size, age, details of the intervention and control groups, assessment measures, follow-up and main results. Of the six RCTs, four used active controls^38^^,^ 39 ^,^ 44 ^,^ 45 and two used the usual treatment of the hemodialysis unit as a control^36^^,^ 37. Only two studies performed follow-up after the intervention, Gross et al. (2017)^38^ with a six-month follow-up and Nejad et al. (2018)^44^ with a one-month follow-up. Most studies excluded participants with suicidal ideation, psychotic disorder, expecting to receive a transplant within three months, and regularly practicing meditation^36^^-^ 38 ^,^ 44 ^,^ 45.

**Table 1:** Characteristics of the randomized clinical trials

			Participants		Intervention	Control			
**Main (year)**	author	**Outline**	**No of participants and no. of participants per group**	**Mean age and SD (years)**	**Format (group por individual), Frequency, program duration**	**Format (group or individual), frequency, duration**	**Post-treatment assessment**	**Measures/Instruments**	**Main results**
Thomas (2017)^37^	et al.	ECR	21 (GI)	66 ± 13 (GI)	Siegal, Williams & Teasdale MBCT, 8 weeks, 3 times a week, lasting between 10-15 minutes, individually next to bed. The participants were encouraged to practice at home in between sessions.	Usual hemodialysis treatment	No follow up	1. Viability:	1.1. 71% retention.
			20 (GC)	64 ± 14 (GC)				1.1 Registration.	1.2. 82% Frequency of all sessions offered.
								1.2 Frequency.	1.3. Good-tolerability meditation, score of 8 on the Likert scale.
								1.3. Tolerability.	
								2. Depression (PHQ-9).	2. With no significant difference between the groups (p = 0.45)
								3. Generalized anxiety disorder (GAD-7)	
									3. With no significant difference between the groups (p = 0.91)

Reilly-Spong (2015)^45^	et al.	ECR with active control	31 (GI)	51.7 ± 12.1 (GI)	tMBSR based on the MBSR program from Kabat-Zinn, Weekly in person group for 5 hours on weeks 1 and 8, and 90-minute teleconferences throughout weeks 2-7, ending with one day of mindfulness retreat on week 8. The participants were encouraged to practice at home between the sessions.	Structured support group, adapted for telephone, facilitated by a life trainer and kidney transplant receiver, teleconference in 90-minute groups, in person, on weeks 1 and 8, and 1 hour throughout weeks 2-7, development of skills with homework included between the sessions.	No follow-up	1.Viability:	1.1. Presence in 84% of the intervention group and 88% in the support group (p = 0,472). With no significant difference between the groups in terms of presence (p = 0.472) and commitment (p > 0.05).
			32 (GC)	53.8 ± 11.4 (GC)				1.1 Presence and commitment	
								1.2 Treatment preference	
								1.3 Satisfaction	
								1.4 Benefit expectation	1.2 Without significant difference in treatment preference (p = 0.340)
								1.5 Treatment loyalty	
									1.3 High satisfaction in the intervention group (8.83) and control group (8.07). With no significant difference between the groups (p = 0.17)



									1.4 Expectation of higher benefits in the intervention group (p = 0.005).

									1.5 All the sessions were held in both groups with small adjustments.

Gross (2017)^38^	et al.	ECR with active control	27 (GI)	52,6 (GI)	tMBSR program based on the MBSR by Kabat-Zinn, 8 weeks, in-person 3-hour workshop on weeks 1 and 8, and 1.5h group teleconference on weeks 2-7. Final 3-hour retreat called “One day of mindfulness”. The participants were encouraged to practice at home between the sessions.	Support group (tSupport) structured, led by a moderator, and designed with the format of the book used by the tMBSR group. Two 1.5-houer workshops and six one-hour weekly workshops. The participants held homework in between the meetings.	6-month follow-up	1. Ansiedade (STAI);	1. No significant difference between the groups after (p = 0.18) and upon the 6-month follow-up (p = 0.55). .
			28 (GC)	54,6 (GC)				2. Depressão (CES-D);	
								3. Qualidade do sono (PSQI);	
								4. Energia/exaustão (PROMIS)	2. Significant difference after (p = 0.05), and no significant difference on the 6-month follow-up (p = 0.86)
								5. Qualidade de vida (MCS e PCS do SF-12)	
								6. Dor (SF-12)	
									3. No significant difference after (p = 0.59) and upon the 6-month follow-up (p = 0.65).
									4. No significant difference after (p = 0.54) and on the 6-month follow-up (p = 0.96).

									5. No significant difference for MCS after (p = 0.34), significant difference for the 6-month follow-up (p = 0.01). No significant difference for PCS after (p = 0.29) and upon the 6-month follow-up (p = 0.96).


									6. No significant difference after (p = 0.99) and upon the 6-month follow-up (p = 0.94).
Solati (2019)^36^ ECR	et al.	ECR	10 (GI)	57 ± 8.32 (GI)	MBCT program from Siegal, Williams and Teasdale, group intervention with 2 to 2.5 hours in each session. The program also includes 45 minutes of daily practice, formal and informal exercises, the participants sometimes record their observations.	Usual treatment of the hemodialysis environment	No follow-up	1. Quality of life (SF-36)	1. Quality of life increased in the GI (p < 0.01). No significant difference between the groups (p > 0.05).
			20 (GC)	60 ± 9.87 (GC)				2. Self-efficacy- General self-efficacy scale	
		
									2. Significant difference for both groups separately (p < 0.01). No significant difference between the groups (p > 0.05).
	
	
Nejad (2018)^44^	et al.	ECR	30 (GI)	55.45 ± 11.6 (Did not specify the age of the groups)	8 mindfulness training sessions; 2 of them in 1.5h sessions and the Other 6 are individual sessions, 30 minutes after the hemodialysis for 1 hour.	8 educational group sessions associated with CKD and hemodialysis, 2 of them were 1.5h group sessions and 6 individual sessions, 30 minutes after hemodialysis for 1 hour.	1 month follow-up	1. General health questionnaire (GHQ-28):	1. Significant difference between the average score in the intervention group after the intervention and on the 1-month follow-up (p < 0.05) for all the GHQ-28 domains. With no significant difference among the groups for all the GHQ-28 domains (p > 0.05).
			30 (GC						
								1.1 Physical symptoms	
								1.2 Anxiety and sleep disorder symptoms	
								1.3 Social functioning failures	
								1.4 Signs of depression	
								1.5 General health	
Park (2014)^39^		ECR, crossover	15	58.7 ± 1.4	The participants heard a prerecorded 14-minute MM using one MP3 player and earphones. The standard recording of the guided meditation included various basic components of mindfulness. There were two to three visits in the early morning.	The participants were submitted to 14 minutes of AP education, listening to a recording on the diagnosis and treatment of hypertension, using the same MP3 player and earphones, in a total of two to three visits in the early morning.	With no follow-up	1. Blood pressure (BP)	1. Significant reduction during MM: SBP (p = 0,004) DBP (p = 0,004) MAP (p = 0,001)
								2. Heart rate (HR)	
								3. Muscle neural sympathetic activity (MNSA)	2. Significant heart rate reduction during the mindfulness meditation (p < 0.001)
								4. Controlled breathing (CB)	
								5. Respiratory rate (RR)	3. Significant MNSA reduction during MM (p = 0.001)

									4. The CB alone did not reduce the BP, HR or MNSA (p = 0.012)

									5. Significant RR reduction during the MM (p < 0.001)


Note. RCT: Randomized clinical trial; IG: Intervention group; CG: Control group; MBCT: Mindfulness-based cognitive therapy; PHQ-9: Patient Health Questionnaire; GAD-7: General Anxiety Disorder; MBRS: Mindfulness-Based Stress Reduction; STAI: State-Trait Anxiety Inventory - state version; CES-D: Epidemiologic Studies Depression Scale; PSQI: Pittsburgh Sleep Quality Index; PROMIS: Fatigue Short Form; SF-12: 12-Item Short-Form Health Survey; MCS: Mental Component Summary; PCS: Physical Component Summary; SF-36: The 36-item Short Form Survey; CKD: Chronic kidney disease; GHQ-28: General Health Questionnaire; eGFR: estimated-Glomerular filtration rate; MM: Mindfulness Meditation; BP: Blood pressure; SBP: Systolic blood pressure; DBP: diastolic blood pressure; MAP: Mean arterial pressure; CB: Controlled breathing; RR: Respiratory rate.

### CHARACTERISTICS OF THE PARTICIPANTS

The six clinical trials involved a total of 264 participants with CKD and a mean age of 57.34 years (± 9.8), ranging from 15 to 63 participants among the studies. Except for the study by Nejad et al. (2018)^44^, that did not inform the gender of the participants, and Park et al. (2014)^39^, which only included male participants, 35.4% of the sample of the remaining included studies were females^36^^-^ 38 ^,^ 45. Data related to demographic status was not well documented; for example, four studies reported the participants' skin color, with an average of 60.2% white and 19.9% ​​black^37^^-^ 39 ^,^ 45. Thomas et al. (2017)^37^^)^ showed that 49% of the participants were married, 50% lived with a family and 46% used psychiatric medications. Four studies described comorbidities associated with CKD, with hypertension and diabetes being the most prevalent^37^^-^ 39 ^,^ 45. Thomas et al. (2017)^47^ presented the results related to comorbidities in more detail, identifying a mean and standard deviation of 10±4, the most prevalent being hypertension, diabetes, dyslipidemia, coronary artery disease, arrhythmias and peripheral vascular disease.

### CHARACTERISTICS OF THE INTERVENTIONS

Most of the studies evaluated used well-established protocols such as mindfulness-based stress reduction (MBSR)^38^^,^ 45 and mindfulness-based cognitive therapy (MBCT)^36^^,^ 37, with some adaptations for the CKD context. Two studies did not report the guiding protocol behind the intervention, but described the practices addressed in the meetings^39^^,^ 44. Apart from one study that carried out two to three individual meetings lasting 14 minutes^39^, the protocols followed an 8-week pattern, with an average duration of 30 minutes to 3 hours per session^36^^-^ 38 ^,^ 44 ^,^ 45.

Two studies adapted the MBSR protocol to be performed through videoconference and in groups (tMBSR), the first and the last face-to-face meeting and the rest were online^38^^,^ 45. Thomas et al. (2017)^37^ held the meetings individually, at the bedside and during the hemodialysis session. Nejad et al. (2018)^44^ also carried out the sessions individually, but the meetings took place after the hemodialysis session.

The practices described in the cited studies ranged from body scanning practices, conscious breathing, raisin practice, gentle arm movement, mindfulness in daily activities and self-compassion, in which participants were invited to adopt a gentle and non-judgmental attitude to respect for experience throughout practices. In addition to the practices carried out in the weekly meetings, most studies encouraged participants to practice at home and keep records over the weeks^36^^-^ 38 ^,^ 44 ^,^ 45.

Thomas et al. (2017)^37^ assessed the feasibility of the intervention through the proportion of eligible participants who enrolled and the proportion of participants who completed the 8-week trial in the intervention group. Of the 20 participants, 15 completed 13 sessions or more and remained until the eighth week with a retention rate of 71%, with the median of the intervention tolerability score equal to 8 out of a total of 10 on the Likert scale^37^. Reilly-Spong et al. (2015)^45^ found that 8 out of 84% of participants in the intervention group attended 3 or more sessions^45^. There was no significant difference between the choices to participate in the active control or intervention group (p=0.340), and in the satisfaction levels for both groups (p=0.17), but future expectations of the benefits of interventions were significantly higher in the intervention group than in the control group (p=0.005)^45^. Two studies reported a total attrition rate of 13% in each group^38^^,^ 45. Thomas et al. (2017)^37^ reported the abandonment of five participants out of a total of 21 before the second session, because they were feeling "clinically very sick" (n=1), "feeling that they had already improved" (n=1) and for "lack of interest" (n=3).

### OUTCOMES

Since this review aims to investigate the impact of interventions based on mindfulness and chronic kidney disease in a broad way, the outcomes found throughout the studies were described as a majority, being mental health outcomes such as depression, anxiety and self-efficacy, physical measures of fatigue, pain, sleep, blood pressure, sympathetic activity, respiratory rate, and psychosocial measures of quality of life (See Table 1).

### MENTAL HEALTH OUTCOMES

#### ANXIETY AND DEPRESSION

Three studies associated the effects of MBIs on anxiety and depression^37^^,^ 38 ^,^ 44. The instruments used for these outcomes varied among studies. For anxiety symptoms, we used the General Anxiety Disorder (GAD-7)^37^, State-Trait Anxiety Inventory - state version (STAI)^38^ and the General Health Questionnaire (GHQ-28) scales, which contains a 6-item subscale for anxiety symptoms and sleep disorders^44^. To measure the depression symptoms, we used the Patient Health Questionnaire (PHQ-9)^37^, Epidemiologic Studies Depression Scale (CES-D)^38^ and the General Health Questionnaire (GHQ-28), which contain a 6-item subscale that measures symptoms of depression^44^. There was no significant difference for both outcomes between the pre- and post-intervention groups in the three studies, and in the one-month^44^ and six-month follow-ups^38^. Although there was no significant difference between groups over time (p>0.05), Nejad et al. (2018)^44^ found significant differences for both anxiety and depression in the intervention group alone, soon after the intervention and at one-month follow-up (p < 0.05). See Table 1.

### SELF-EFFICACY

Solati et al. (2019)^36^ reported self-efficacy using the General Self-efficacy Scale questionnaire. There was no significant difference between the two groups after the intervention (p > 0.05), but intragroups, the mean self-efficacy score increased by 0.95 in the control group and 5.2 points in the intervention group, with a significance level of p < 0,01^36^ (See Table 1).

### PHYSICAL MEASURES

#### FATIGUE AND PAIN

Gross et al. (2017)^38^ measured fatigue and pain using the PROMIS-Fatigue Short Form v1.0 and 12-Item Short-Form Health Survey (SF-12) scales in the item related to pain interference, but the authors did not identify significant differences for both the outcomes after the intervention (p > 0.05) and over time in the six-month follow-up (p > 0.05)^38^ (See Table 1).

#### SLEEP

Two studies assessed sleep quality. The authors used different scales to assess this outcome. Gross et al. (2017)^38^ used The Pittsburgh Sleep Quality Index (PSQI)^38^ scale, while Nejad et al. (2018)^44^ measured using the General Health Questionnaire (GHQ-28), which contains a 6-item subscale for symptoms of anxiety and sleep disorders^44^. Nejad et al. (2018)^44^ found a significant difference in the intervention group alone soon after the intervention, and at the one-month follow-up (p < 0,05)^44^, but in both studies, there were no significant results between the groups after the intervention and in the one- and six-month follow-ups (p > 0.05)^38^^,^ 44 (See Table 1).

## BLOOD PRESSURE, SYMPATHETIC ACTIVITY AND RESPIRATORY RATE

Park et al. (2014)^39^ assessed blood pressure, respiratory rate and sympathetic activity during mindfulness practice. Blood pressure was measured using an automated sphygmomanometer (Dinamap PRO Series), sympathetic activity was measured directly in the peroneal nerve by microneurography, and for breathing, the participants were instructed to maintain a respiratory rate of 12 breaths/min. There were significant differences between the groups after the intervention for all outcomes during meditative practice (p < 0.05) (See Table 1).

### PSYCHOSOCIAL MEASURES

#### QUALITY OF LIFE

Two studies assessed quality of life using different scales. Solati et al. (2019)^36^ assessed this outcome using the 36-item Short Form Survey (SF-36), while Gross et al. (2017)^38^ measured through the physical and mental component of The Short Form-12v2 (SF-12) scale. One study found a significant difference in the mental component related to quality of life between the groups only in the six-month follow-up (p = 0.01), but there was no significant difference in the physical component (p > 0.05)^38^. Solati et al. (2019)^36^ did not find significant results between the groups (p > 0.05); however, when analyzed alone within groups, there was a significant difference in the improvement of quality of life in the intervention group after the intervention (p < 0.01) (See Table 1).

### METHODOLOGICAL QUALITY OF THE INCLUDED STUDIES

The quality of the studies was measured using The Risk of Bias 2 (RoB 2), in its updated version^43^ for the 6 studies. Regarding the randomization process, two studies adequately described the methodology implemented to generate randomization and the blinding of the researchers^37^^,^ 45. For the domain of interventions, three studies cited the blinding of participants in relation to the allocated interventions (in studies with active controls), power of effect, and found no evidence of contamination between the groups^37^^,^ 38 ^,^ 45. Most studies described the missing results^37^^,^ 38 ^,^ 44 ^,^ 45, except for two studies in which the authors do not mention the exclusion of some results^36^^,^ 39.

**Figure 2:**
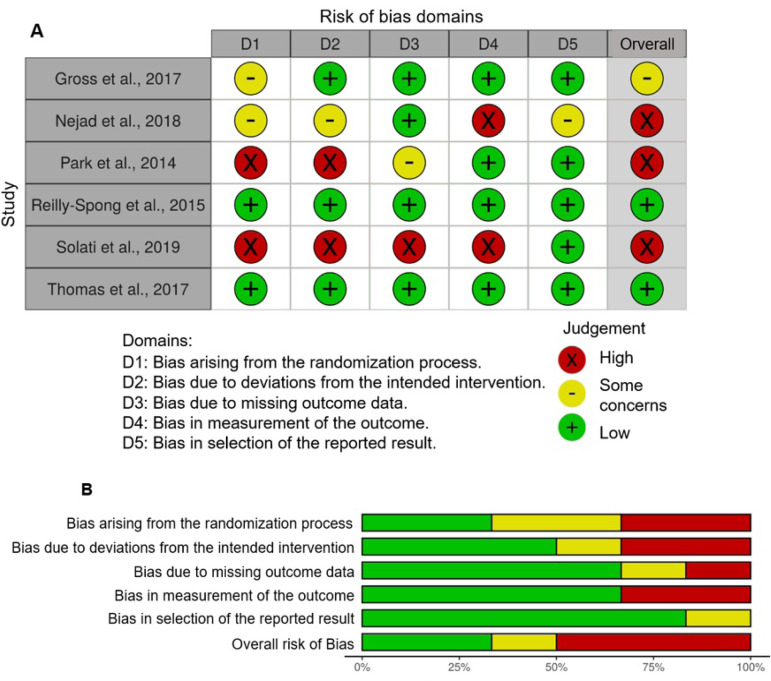
Assessment of the risk of bias of the included studies Note. A: Summary of risk of bias for each trial; B: Graph on each risk of bias presented as percentages across all included studies.

In the domain referring to the measurement of outcomes, all studies cited the psychometric properties of the instruments used, as well as the justification for implementing the adopted measures. Only two studies did not have enough information about the outcome and whether the intervention could have been interfered from the responses^36^^,^ 44. Regarding the selection of reported results, only Nejad et al. (2018)^44^ did not report the analysis plan for the outcomes found^44^. Finally, only two studies can be considered of high methodological quality^45^^,^ 47 (See Figure 2).

## DISCUSSION

MBIs have grown worldwide as a complementary therapy in the treatment of chronic diseases, including CKD^24^^,^ 28 ^,^ 40. To our knowledge, this may be the first systematic review to comprehensively assess the existing literature on the impacts of MBIs on adults with CKD undergoing hemodialysis. Six studies eligible for inclusion were identified, which varied in nature. Four studies used active controls,^38^^,^ 39 ^,^ 44 ^,^ 45 and only two evaluated follow-ups after the intervention^38^^,^ 44. Only three studies showed adequate sample size, effect power and samples^37^^,^ 38 ^,^ 45 ^.^.^)^ The attrition rate was described in two studies, being 13% for each group^38^^,^ 45. MBIs's protocols were not addressed homogeneously among the papers. Two studies used the MSBR protocol, but it was adapted to be performed by videoconference (tMBSR)^38^^,^ 45; two others use the MBCT protocol, also with adaptations for the context of hemodialysis^36^^,^ 37.

The results of the six studies are encouraging for the domains of mental health, physical measures and quality of life. There were significant improvements in the symptoms of anxiety, depression, greater self-efficacy, sleep quality and quality of life in the groups submitted to the intervention^36^^,^ 38 ^,^ 44. Similar results were found in other studies that evaluated the effects of MBIs on chronic diseases such as diabetes^46^, HIV^47^, irritable bowel syndrome^48^, chronic insomnia^49^ and recurrent episodes of depression^50^. Such results may be due to the fact that mindfulness practices are related to cognitive changes in patterns of actions, thoughts and emotions, increasing awareness of psychological and physical states, with greater openness and without judgment^21^^,^ 22 ^,^ 51. By adopting and cultivating this attentive posture throughout the practices, such as conscious breathing and body scanning, it is possible to understand that painful sensations or situations and negative emotions do not need to be fought or silenced to live an expressive life^27^.

In addition to psychological and psychosocial stressors, the presence of comorbidities associated with CKD, such as hypertension, is very prevalent in people with CKD^52^. Previous studies found significant results, like those reported by Park et al. (2014)^39^ on the effects of mindfulness meditations on the physical measures of blood pressure, heart rate, and respiratory rate^53^^-^ 56. One of the potential mechanisms behind these results may be the fact that MBIs are associated with a reduction in sympathetic activity via an inflammatory decrease, mainly acting on markers such as C-reactive protein (CRP), tumor necrosis factor-alpha (TNF-α) and interleukin 8 (IL-8) described in previous studies^57^^,^ 58.

This review enabled a broad understanding of the impacts of MBIs on CKD patients undergoing hemodialysis and employed a rigorous methodological strategy to research and evaluate the literature on this topic. Two reviewers were involved in the screening and evaluation of studies eligible for inclusion, with in-depth discussions regarding the proposed evaluations, and with the help of a third reviewer, when necessary. The methodological quality of the studies was carefully assessed according to the recommendations in the Cochrane Manual (Cochrane Collaboration 2020)^43^.

The fact that MBIs originate from ancient Eastern traditions, and the resource constraints for translations in languages ​​other than English, may have biased our findings. In addition, the low methodological quality of three studies together and the general heterogeneous nature of the evaluations/outcomes of the analyzed papers made it impossible to carry out a quantitative meta-analysis.

Reilly-Spong et al. (2015)^45^, Gross et al. (2017)^38^ and Thomas et al. (2017)^37^ produced well-designed RCTs with adequate numbers of participants per group based on sampling power^37^^,^ 38 ^,^ 45. Its strict inclusion and exclusion criteria, and the use of MBIs protocols consolidated in the literature, carried out by certified and experienced professionals in the field, and with assessments ranging from pre-post to 6-month follow-up, allow a high level of confidence when reviewing its outcomes^37^^,^ 38 ^,^ 45. Four studies used active controls faithful to the dynamics presented in the groups that received the mindfulness intervention, and did not document contamination between the groups^38^^,^ 39 ^,^ 44 ^,^ 45. These findings are in line with the guidelines suggested in a recent narrative review study regarding mindfulness practices and CKD^40^.

In some studies, the authors used instrument subscales to assess their secondary outcomes, such as the item related to pain inserted in the SF-12^38^ scale, and the subscales for anxiety and depression present in the GHQ-28^44^ scale. In the study by Nejad et al. (2018)^44^ the same subscale inserted in the GHQ-28 scale measured two different outcomes (anxiety and sleep disorders) for the same domain, containing only 6 questions for these outcomes. We believe that the use of these subscales may have compromised the sensitivity in assessing the variable of interest^44^. None of the studies assessed the participants' level of mindfulness, such measurement could guide researchers regarding the skills developed throughout the program and broaden the discussion of the results.

Although most of the papers evaluated used MBIs based on well-established protocols and certified mindfulness instructors, two studies did not specify the information on the protocol base of the program adopted, making it difficult to generalize the results^39^^,^ 44; in addition, Park et al. (2014)^39^ held only three brief meetings during the week the participants underwent hemodialysis^39^. Finally, three studies presented weak methodologies, not clearly describing the randomization processes, the excluded results and the applied intervention. Therefore, the results of these studies should be treated with caution^36^^,^ 39 ^,^ 44.

Future studies involving MBIs in patients with CKD should be carried out on larger scales, and with the implementation of robust methodologies; examine both physical and psychological measures, quantitatively or qualitatively, in order to further explore the clinical implications of interventions in this population. The sociodemographic characteristics, disease stages and associated comorbidities, sample stratifications, and issues regarding the necessary adaptations for the application of appropriate MBIs to the hemodialysis context, whether individually, in groups or online.

## CONCLUSIONS

Although the evidence is limited, this review indicates that MBIs may offer a promising, safe and non-invasive complementary therapy for patients with CKD on hemodialysis, specifically in relation to mental health, quality of life and the physical aspects of the disease. The implementation of these interventions must consider the certification of instructors and details of the protocols, ensuring their reliability. The potential impacts of MBIs for people with CKD require studies with higher methodological quality, clarifying the feasibility of different formats of interventions presented, and long-term evaluations.
